# Human induced pluripotent stem cells‐derived liver organoids grown on a Biomimesys® hyaluronic acid‐based hydroscaffold as a new model for studying human lipoprotein metabolism

**DOI:** 10.1002/btm2.10659

**Published:** 2024-03-16

**Authors:** Meryl Roudaut, Amandine Caillaud, Zied Souguir, Lise Bray, Aurore Girardeau, Antoine Rimbert, Mikaël Croyal, Gilles Lambert, Murielle Patitucci, Gaspard Delpouve, Élodie Vandenhaute, Cédric Le May, Nathalie Maubon, Bertrand Cariou, Karim Si‐Tayeb

**Affiliations:** ^1^ Nantes Université, CHU Nantes, CNRS, Inserm, l'institut du thorax Nantes France; ^2^ HCS Pharma Lille France; ^3^ CRNH‐Ouest Mass Spectrometry Core Facility Nantes France; ^4^ Inserm, UMR 1188 Diabète Athérothrombose Thérapies Réunion Océan Indien (DéTROI) Université de La Réunion Saint‐Denis de La Réunion France

**Keywords:** cytochrome activities, hiPSC‐derived liver organoids, hyaluronic‐acid based hydroscaffold, lipid metabolism, liver steatosis, Lp(a)

## Abstract

The liver plays a key role in the metabolism of lipoproteins, controlling both production and catabolism. To accelerate the development of new lipid‐lowering therapies in humans, it is essential to have a relevant in vitro study model available. The current hepatocyte‐like cells (HLCs) models derived from hiPSC can be used to model many genetically driven diseases but require further improvement to better recapitulate the complexity of liver functions. Here, we aimed to improve the maturation of HLCs using a three‐dimensional (3D) approach using Biomimesys®, a hyaluronic acid‐based hydroscaffold in which hiPSCs may directly form aggregates and differentiate toward a functional liver organoid model. After a 28‐day differentiation 3D protocol, we showed that many hepatic genes were upregulated in the 3D model (liver organoids) in comparison with the 2D model (HLCs). Liver organoids, grown on Biomimesys®, exhibited an autonomous cell organization, were composed of different cell types and displayed enhanced cytochromes P450 activities compared to HLCs. Regarding the functional capacities of these organoids, we showed that they were able to accumulate lipids (hepatic steatosis), internalize low‐density lipoprotein and secrete apolipoprotein B. Interestingly, we showed for the first time that this model was also able to produce apolipoprotein (a), the apolipoprotein (a) specific of Lp(a). This innovative hiPSC‐derived liver organoid model may serve as a relevant model for studying human lipopoprotein metabolism, including Lp(a).

**Translational Impact Statement**

The hiPSC‐derived liver organoids, grown on hyaluronic acid‐based hydroscaffold Biomimesys®, is an innovative method that recapitulates in vitro the entire metabolism of lipoproteins, including the secretion of Lp(a). This model also offers interesting functionality for toxicology and nonalcoholic fatty liver disease studies and will allow the characterization of new genes as potential therapeutic targets in vitro.

## 1 INTRODUCTION

Patient‐specific induced pluripotent stem cell (iPSC)‐derived hepatocyte‐like cells (HLCs) is a renewable source of hepatocytes that can be obtained in a noninvasive way. This model was previously described as a tool for disease modeling and drug screening.^1^ For example, it was found suitable for recapitulating the pathophysiological impact of specific rare genetic variants in hereditary metabolic diseases or familial dyslipidemias observed in patients.^2^, ^3^ However, applications of this model for drug development has been strongly limited by the fact that HLCs are more closely related to fetal rather than mature hepatocytes.^4^

In order to improve the functionality of these in vitro models, Clevers and Sato first introduced in 2009 the concept of “organoids,” highlighting the importance of the microenvironment for the improvement of stem‐cell derived models.^5^ Later, the Takebe's team, in addition to the introduction of supportive cell lineages in coculture, confirmed that the functionality of hiPSC‐derived liver models was improved when hepatocytes were self‐organized in three‐dimensional (3D).^6^ Since then, other 3D differentiation protocols have been described and confirmed the need of a matricial microenvironment to potentiate the hiPSC aggregation and differentiation in 3D.^7^, ^8^, ^9^, ^10^

Various 3D cell culture systems have been used for mimicking the native extracellular matrix (ECM), like hydrogels and solid scaffolds.^11^, ^12^ For example, the Matrigel™ is the most widely used product for the self‐organization and differentiation of organoids.^7^, ^9^, ^10^ However its use remains limited to specific research applications due to batch‐to‐batch variability and to its animal‐derivative origin.^13^ Therefore, there is still an unmet need for the development of synthetic defined matrices mimicking at the best the native ECM, that supports the culture and differentiation of hiPSCs for further preclinical applications. In this context, decellularized tissues might appear as the gold standard for reproducing the ECM, but they suffer low availability, high technicity, and batch‐to‐batch variability.^14^

Here, we propose the use of Biomimesys® as an innovative hydroscaffold for hiPSC aggregation and differentiation toward liver organoids. This matrix is a highly porous (between 50 and 200 μm) animal free synthetic hydroscaffold, composed of hyaluronic acid (HA) enriched with collagen fibers and Arginylglycylaspartic acid (RGDS) adhesion peptide. The differentiation process within this matrix was optimized from a previous protocol^15^ for its application in 3D and improved for the generation of other supportive lineages. Compared to previously published protocols, this 28‐day protocol is a direct patterning of hiPSCs toward liver organoids with no 2D intermediate steps before the 3D patterning, and no addition of exogenous supportive lineages.

The resulting 3D model was characterized in order to confirm the presence of functional hepatic cells and supportive lineages. Transcriptomic and functional analysis were performed to compare liver organoids with HLCs in order to confirm the value of this 3D model for the study of specific liver functions such as lipoprotein or xenobiotics metabolism.

As a proof of concept of the functional gain of this model, we tested the production of apolipoprotein (a) (Apo(a)) which is a protein constitutive of the lipoprotein Lp(a). Lp(a) is an independent risk factor for atherosclerotic cardiovascular diseases and the target of novel Lp(a)‐lowering therapies that block the hepatic production of Apo(a).^16^ One of the major problems in studying the biology of Lp(a) is the fact that no in vitro models except primary human hepatocytes (PHH) produce native Apo(a). To address this issue, we generated hiPSCs from a patient carrying genetically high levels of Lp(a)^17^ and tested Apo(a) production in 2D versus 3D conditions.

## 2 RESULTS

### 2.1 An ECM for hiPSC‐derived liver organoid formation

**FIGURE 1:**
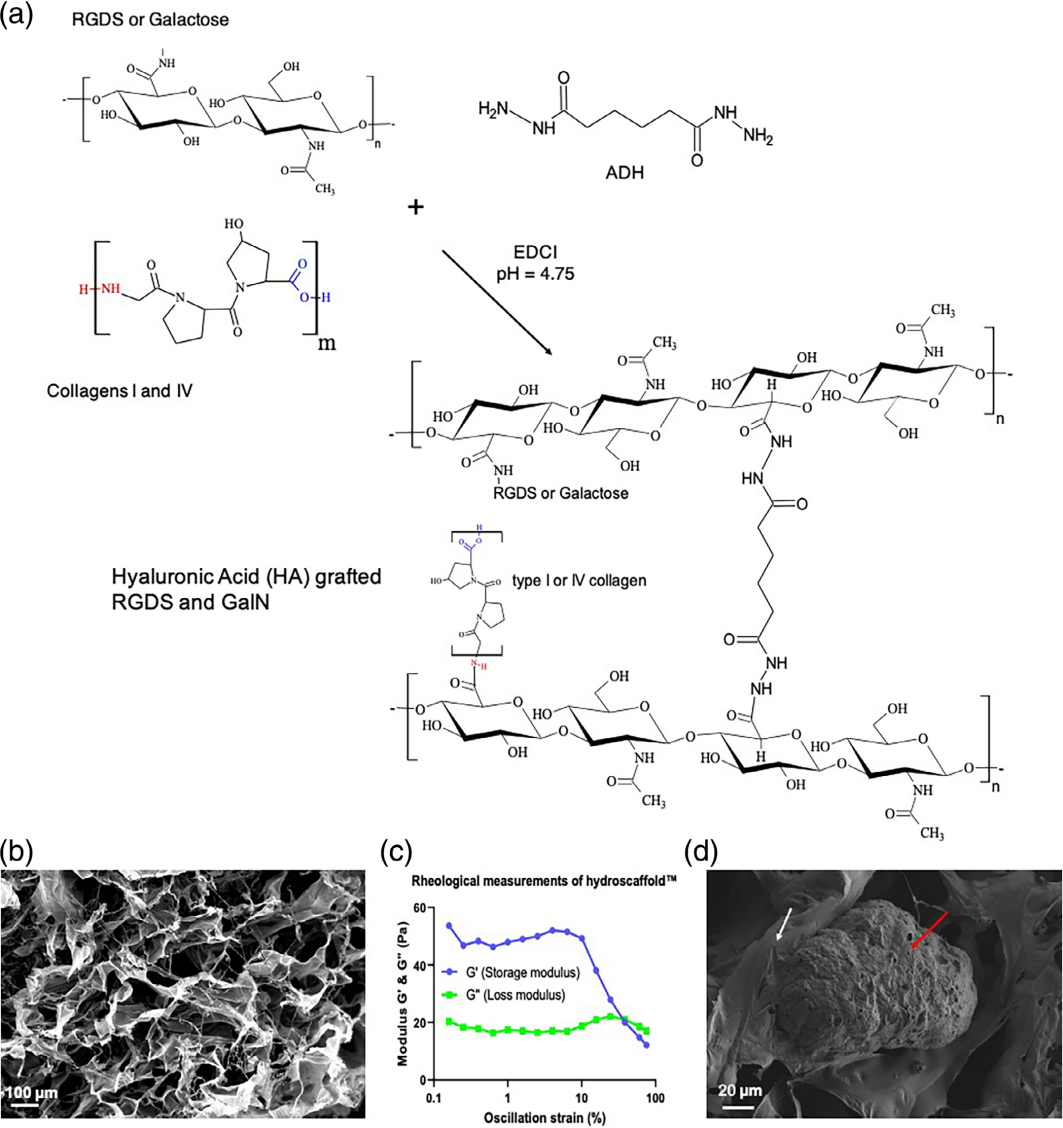
A 3D environment for hiPSC differentiation into liver organoids. (a) Step and formulation for Hydroscaffolds™ production. (b) Scanning electron microscopy (SEM) observations of the dehydrated scaffold; scale bar = 100 μm. (c) Rheological measurements of HA‐g‐RGDS and type I and IV collagens: storage modulus (*G*') (blue) and loss modulus (*G*″) (green); *T* = 37°C, frequency = 1 Hz. (d) SEM observations of the dehydrated scaffold (white arrow) in culture with liver organoid (red arrow); scale bar = 20 μm.

To mimic the liver ECM, we developed a hydroscaffold made of chemically modified HA grafted with RGDS and combined with two major collagens found in the liver ECM^18^ (Figure 1a). These type I and IV collagens, at, respectively, 7 and 3.3 μg/ml were introduced in physiological amount.^19^ The scanning electron microscopy (SEM) analysis of the lyophilized hydroscaffold showed the presence of collagen fibers and a porosity modulated by HA sheets (Figure 1b). This porosity was ranging between 50 and 200 μm and makes the diffusion of the cells inside the matrix easier.

Rheological analysis of hydrated matrix was performed 1 week after cell culture and different parameters were evaluated in order to characterize the hydroscaffolds™: the storage modulus (*G*′), the loss modulus (*G*″), and the deformation (*γ*) (Supplemental Materials and Methods, Figure 1c). We observed that the storage modulus is larger than the loss modulus which is indicative of a viscoelastic solid behavior. At low deformation (< 10%) *G*′ and *G*″ remain constant, indicating that the hydroscaffolds structure is undisturbed. This region is called the linear viscoelastic. We observed that as soon as the moduli started to decrease, the structure was disturbed.

Then, we measured an elastic modulus at 0.15 ± 0.05 kPa and a swelling ratio at 60 ± 10 g/g, which are indicative of a soft matrix with an elastic modulus nearly similar to the modulus of decellularized liver or ECM liver.^20^ The high mechanical stability of Biomimesys® makes this matrix suitable for a long‐term cell culture in the same hydroscaffold™. Furthermore, we observed a direct interaction between the hydroscaffold and liver organoid using electron microscopy (Figure 1d).

### 2.2 Generation of a hiPSC‐derived liver organoid model

**FIGURE 2:**
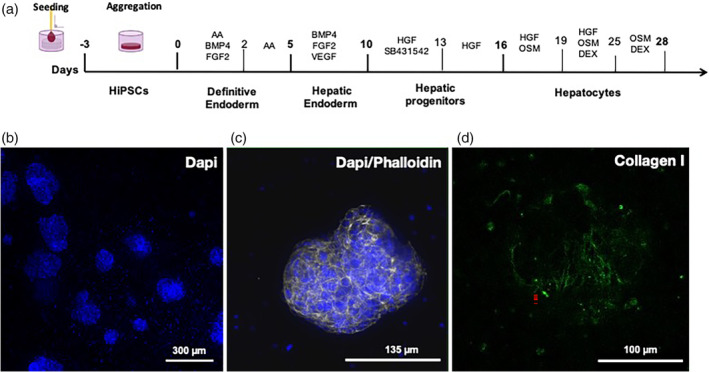
HiPSCs differentiation protocol in Biomimesys® Liver for the generation of liver organoids. (a) Schematic diagram of the hiPSC differentiation protocol into liver organoids. (b) DAPI staining of liver organoids at low magnification (overall view of a 96‐well plate). (c) Actin filament staining in liver organoids using phalloidin‐488 (green). (d) Collagen type I fibers (blue) visualized using bi‐photonic microscopy in a liver organoid.

The differentiation protocol was optimized from the one set up in 2010 by Stephen Duncan's team,^15^ where different cytokines were sequentially used to mimic the different stages of the liver development (Figure 2, Table 1). In order to gain in maturity, 3D cell culture conditions were setup with the use of Biomimesys® as a microenvironment for cell aggregation and direct differentiation. HiPSCs were exposed to the same set of cytokines as described previously^15^ with increased concentrations and/or incubation times, including Activin A, bone morphogenetic protein 4, fibroblast growth factor 2 (FGF2), hepatocyte growth factor (HGF), and oncostatin M (OSM). In addition, and in order to improve the initial protocol, other molecules were added to potentiate the effect of some cytokines during the different phases of differentiation (vascular endothelial growth factor [VEGF], SB431542, and dexamethasone). It is known that FGF2, used during the specification of the endoderm into the liver endoderm through the activation of Naked cuticle 1 (NKD1), will inhibit the Wnt signaling pathway.^21^ On the one hand, VEGF has been shown to be an effective molecule which enhances the differentiation step through NKD1 activation and via Rac1 GTPase.^22^ On the other hand, the combination of SB431542, an inhibitor of the transforming growth factor beta pathway^23^ and HGF, an activator of the Wnt pathway^24^ resulted in an improved differentiation into hepatoblasts. Finally, we also used dexamethasone, a molecule known to enhance hepatocyte maturation by arresting hepatocyte proliferation and to induce the expression of numerous transcription factors and cellular pathways.^25^ This molecule is used in many differentiation protocols and we observed that its use in synergy with OSM resulted in enhanced expression of hepatocyte genes such as albumin (*ALB*), apolipoprotein B (*APOB*), proprotein convertase subtilisin/kexin type 9 (*PCSK9*), and the cytochrome P450 type 3A4 (CYP3A4) (*data not shown*).

**TABLE 1:** Three‐dimensional differentiation protocol of hiPSC in Biomimesys®.

Days^45^	Incubation conditions	Milieu	Cytokines/molecules
D‐3	Hypoxia (4% O_2_, 5% CO_2_)	StemMACS™ iPS‐Brew XF medium (Miltenyi)	ROCK inhibitor (Y27632, 10 μM) (Cell Guidance Systems)
D‐2		StemMACS™ iPS‐Brew XF medium (Miltenyi)	‐
D‐1		‐	‐
D0	Normoxia (20% O_2_, 5% CO_2_)	RPMI 1640 medium (Life Technologies) + B27 (with insulin) (Life Technologies)	Activin A (100 ng/ml) (Miltenyi) FGF2 (40 ng/ml) (Miltenyi) BMP4 (20 ng/ml) (Miltenyi)
D1		‐	‐
D2		RPMI 1640 medium (Life Technologies) + B27 (with insulin) (Life Technologies)	Activin A (10,000 ng/ml) (Miltenyi)
D3		‐	‐
D4		‐	‐
D5	Hypoxia (4% O_2_, 5% CO_2_)	RPMI 1640 medium (Life Technologies) + B27 (with insulin) (Life Technologies)	VEGF (200 ng/ml) (Miltenyi) FGF2 (50 ng/ml) (Miltenyi) BMP4 (100 ng/ml) (Miltenyi)
D6		‐	‐
D7		RPMI 1640 medium (Life Technologies) + B27 (with insulin) (Life Technologies)	VEGF (200 ng/ml) (Miltenyi) FGF2 (50 ng/ml) (Miltenyi) BMP4 (100 ng/ml) (Miltenyi)
D8		‐	‐
D9		‐	‐
D10		RPMI 1640 medium (Life Technologies) + B27 (with insulin) (Life Technologies)	HGF (100 ng/ml) (Miltenyi) SB431542 (10 μg/ml) (Cell Guidance Systems)
D11		‐	‐
D12		‐	‐
D13		RPMI 1640 medium (Life Technologies) + B27 (with insulin) (Life Technologies)	HGF (100 ng/ml) (Miltenyi)
D14		‐	‐
D15		‐	‐
D16		RPMI 1640 medium (Life Technologies) + B27 (with insulin) (Life Technologies)	HGF (100 ng/ml) (Miltenyi) OSM (20 ng/ml) (Miltenyi)
D17		‐	‐
D18		‐	‐
D19	Normoxia (20% O_2_, 5% CO_2_)	Hepatocyte culture medium (HCM) (Lonza) without EGF	HGF (100 ng/ml) (Miltenyi) OSM (200 ng/ml) (Miltenyi) DEX (0,5 μM) (SIGMA)
D20		‐	‐
D21		‐	‐
D22		Hepatocyte culture medium (HCM) (Lonza) without EGF	HGF (100 ng/ml) (Miltenyi) OSM (200 ng/ml) (Miltenyi) DEX (1 μM) (SIGMA)
D23		‐	‐
D24		‐	‐
D25		Hepatocyte culture medium (HCM) (Lonza) without EGF	OSM (200 ng/ml) (Miltenyi) DEX (1 μM) (SIGMA)
D26		‐	‐
D27		‐	‐
D28		Hepatocyte culture medium (HCM) (Lonza) without EGF	‐

During the differentiation process, cell‐clusters form randomly in the hydroscaffold. It is noticeable that the cell clusters, hereafter referred to as liver organoids, are preferentially distributed in the periphery of the hydroscaffold (Figure 2b). Following labeling of the cytoskeleton with phalloidin, we observed that liver organoids form cavities within self‐organized cells (Figure 2c) and produce collagen I fibers (Figure 2d).

### 2.3 Transcriptomic analysis of liver organoids

**FIGURE 3:**
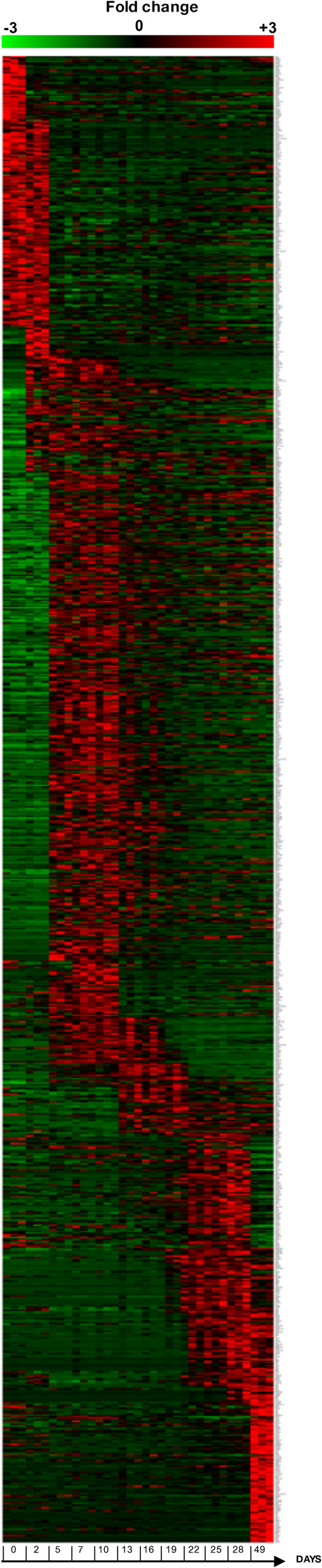
HiPSC‐liver organoids characterization by 3′SRP transcriptional analysis. Heatmap displaying specific genes expression at different time points of differentiation (0, 2, 5, 7, 10, 13, 16, 19, 22, 25, and 28 days) and 3 weeks later (49 days total) (*n* = 3 independent differentiations) with a two folds greater expression compared to previous and following time points (*p* < 0.05).

**FIGURE 4:**
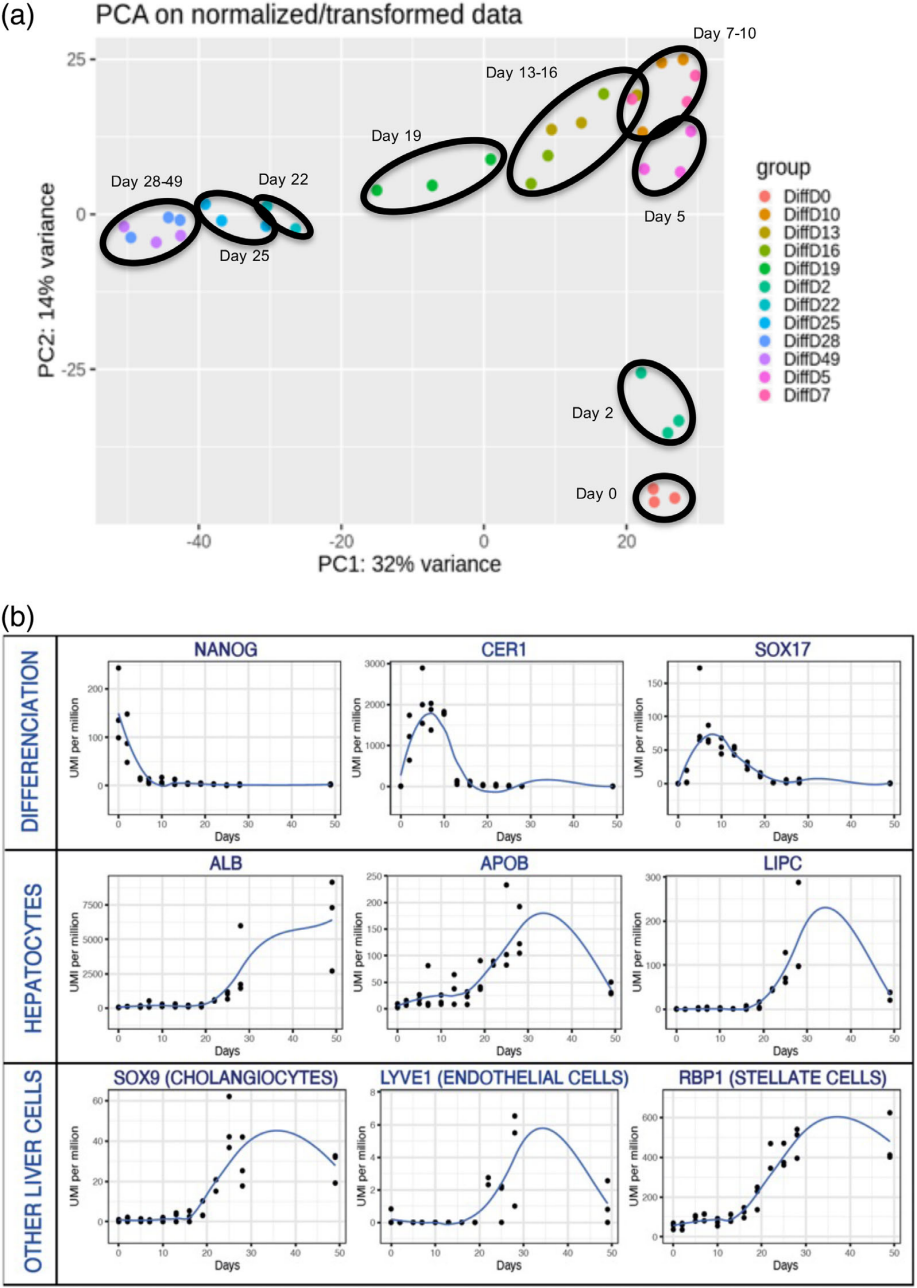
3′SRP transcriptional analysis. (a) Principal component analysis (PCA) throughout liver organoids differentiation showing time point clustering (black circles). (b) Genes specifically expressed during the differentiation (*NANOG*, *CER1*, *SOX17*), in hepatocytes (*ALB*, *APOB*, *LIPC*), and in other liver cells (*SOX9*, *LYVE1*, *RBP1*).

3′SRP was performed at different time points during differentiation (days 0, 2, 5, 7, 10, 13, 16, 19, 22, 25, 28, and 49). This allowed us to observe differentially expressed genes during liver organoids differentiation (Figure 3). The principal component analysis performed on the 3′SRP transcriptional data showed a good clustering of our different differentiations and a good separation of the different time points, with the exception of days 7 and 10 (Figure 4a). Further analyses showed a sequential expression of differentiation markers such as *NANOG* on days 0–2; *CER1* (Cerberus 1) on days 2–10 and *SOX17* on days 5–19 (Figure 4b). Specific hepatic markers such as *ALB*, *APOB*, and hepatic lipase (*LIPC*) displayed an increased expression from day 19 to day 35. However, we observed a decrease of the hepatic gene expression beyond day 35, highlighting the need to improve culture conditions for a long‐term maintenance of liver organoids. Interestingly, we observed that markers specific for supportive lineage such as *SOX9*, *LYVE1*, and *RBP1* were expressed on days 19–35, confirming the multicellular nature of liver organoids. Most of the transcriptomic results were confirmed by qPCR analysis in multiple organoid differentiation experiments (Supplemental Figure 1).

Subsequently, and to better characterize the model, RNAseq was performed on the liver organoid and HLC models at the beginning (day 0) and at the end of differentiation (day 20 for HLCs and day 28 for liver organoids) (Supplemental Materials and Methods, Supplemental Figure 2). The first data showed little difference in early differentiation for 2D and 3D hiPSCs, but many differences in gene expression at the end of the differentiation between HLCs and liver organoids (Supplemental Figure 2a). Indeed, 7448 out of 57,905 genes were differentially expressed between the 2D HLC and 3D liver organoids model (Supplemental Figure 2b). Among differentially expressed genes, genes associated with extracellular functions were more expressed in liver organoids model than in HLCs (Supplemental Figure 3). It is also important to note that the expression of markers of apoptosis and cell viability remained stable during the differentiation of our model (Supplemental Figure 4).

### 2.4 Phenotypic characterization of liver organoids

**FIGURE 5:**
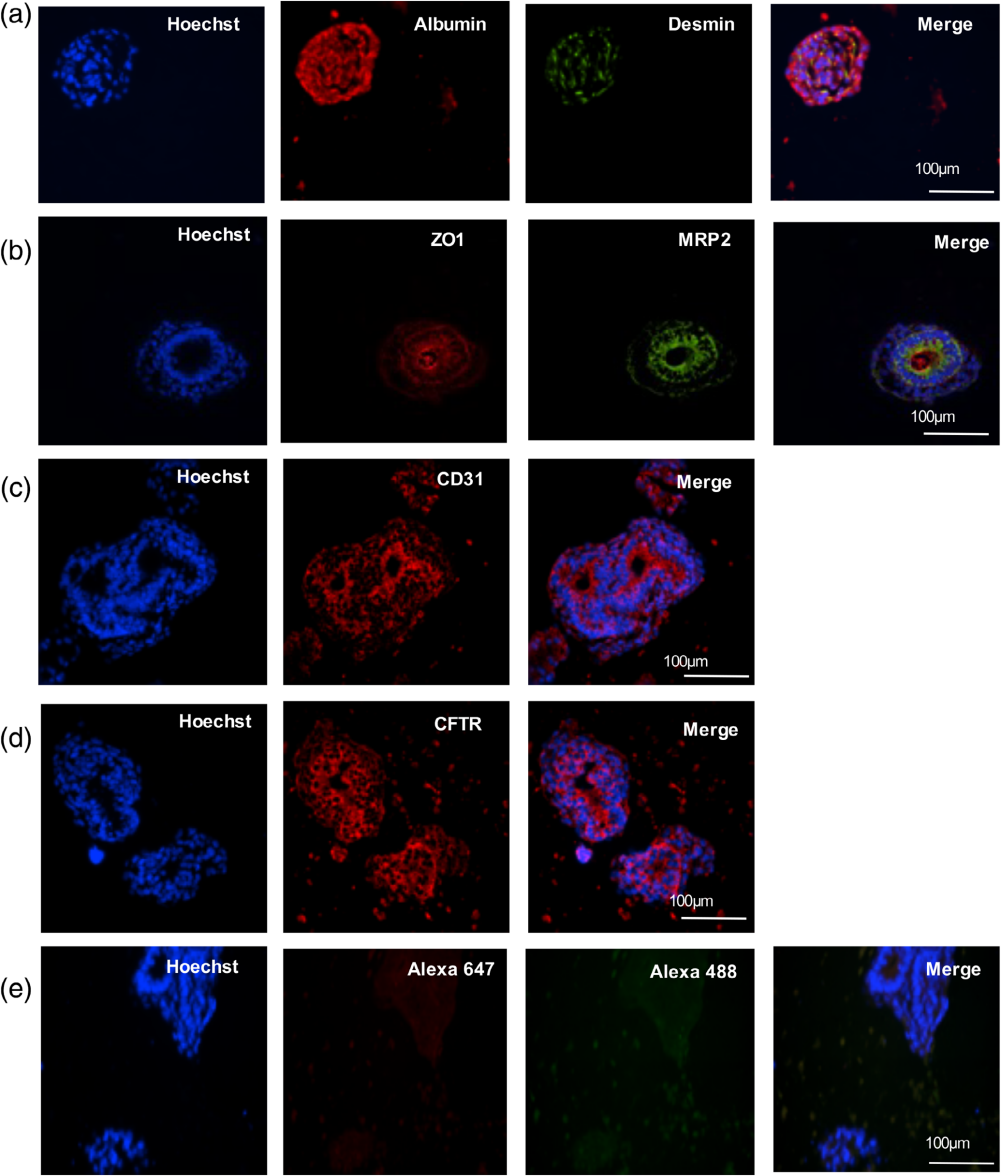
Liver organoids' characterization by immunostaining. Liver organoids were stained in addition to Hoechst (nuclei, blue) for the following makers. (a) Albumin (red), desmin (green). (b) Zonula Occludens‐1 (ZO‐1) (red), multidrug resistance‐associated protein 2 (MRP2) (green). (c) CD31 (red). (d) CFTR (red). (e) Negative control without primary antibody. Scale bar = 100 μm.

The presence of different cell populations within the liver organoids was examined by immunofluorescence. First, the presence of hepatocytes was confirmed using albumin staining (Figure 5, panel a). The polarization of the liver organoids was evidenced by the presence of intercellular junction proteins such as Zonula Occludens‐1. Also, their ability to metabolize xenobiotics was confirmed using multidrug resistance‐associated protein 2 transporters staining (Figure 5, panel b). Stellate‐cells were visualized using desmin staining (Figure 5, panel a), while sinusoidal endothelial cells and cholangiocytes were, respectively, identified using CD31 and CFTR staining (Figure 5, panels c and d). Unspecific labeling was controlled using liver organoids only stained with corresponding secondary antibodies only (Figure 5, panel e).

### 2.5 Functional characterization of liver organoids

#### 2.5.1 Xenobiotic metabolism

**FIGURE 6:**
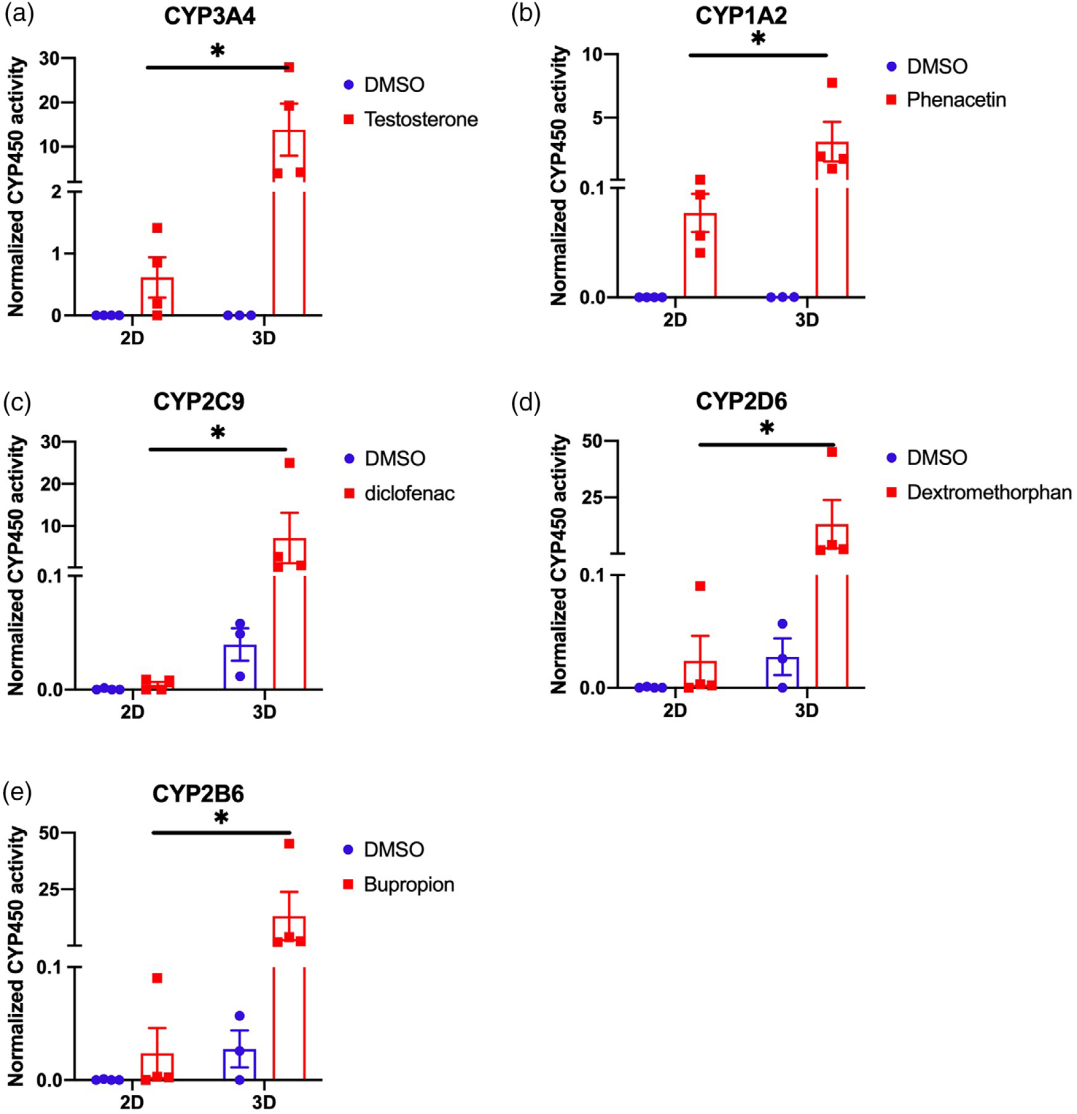
CYP450 activity in hepatocyte‐like cells (HLCs) (2D) and liver organoids (3D) measured in basal conditions and after induction with either DMSO (vehicle control), testosterone (CYP3A4) (a), phenacetin (CYP1A2) (b), diclofenac (CYP2C9) (c), dextromethorphan (CYP2D6) (d), and bupropion (CYP2B6) (e) treatments. Values are normalized against total RNA content (*n* = 4 independent differentiations). Statistical significance was assessed using unpaired *t* test, with a *p* value cut‐off set at *p* < 0.05. *, *p* value <0.05; **, *p* value <0.01; ***, *p* value <0.001; ns, not significant.

In vivo‐like in vitro investigation on drug toxicity is one of the main expectations of the liver organoid model, in order to offer a potential alternative to pharmacodynamic studies in humans.^26^ The activities of 5 cytochrome (CYPs) enzymes (3A4, 1A2, 2C9, 2D6, 2B6), which represent the majority of cytochrome families (CYP1, CYP2, and CYP3) expressed in the liver,^27^ were tested in differentiated liver organoids and HLCs after exposure to xenobiotics (the detailed list of the products and concentrations is described in Supplemental Material and Methods section). As shown in Figure 6, the induction of CYP activity of each tested isoforms was significantly increased in fully differentiated 3D liver organoids (day 28) when compared to differentiated 2D HLCs (day 20). Taken together, these results show that the 3D‐liver organoid model is more functional than HLCs for studying drug metabolism.

#### 2.5.2 Lipid metabolism

**FIGURE 7:**
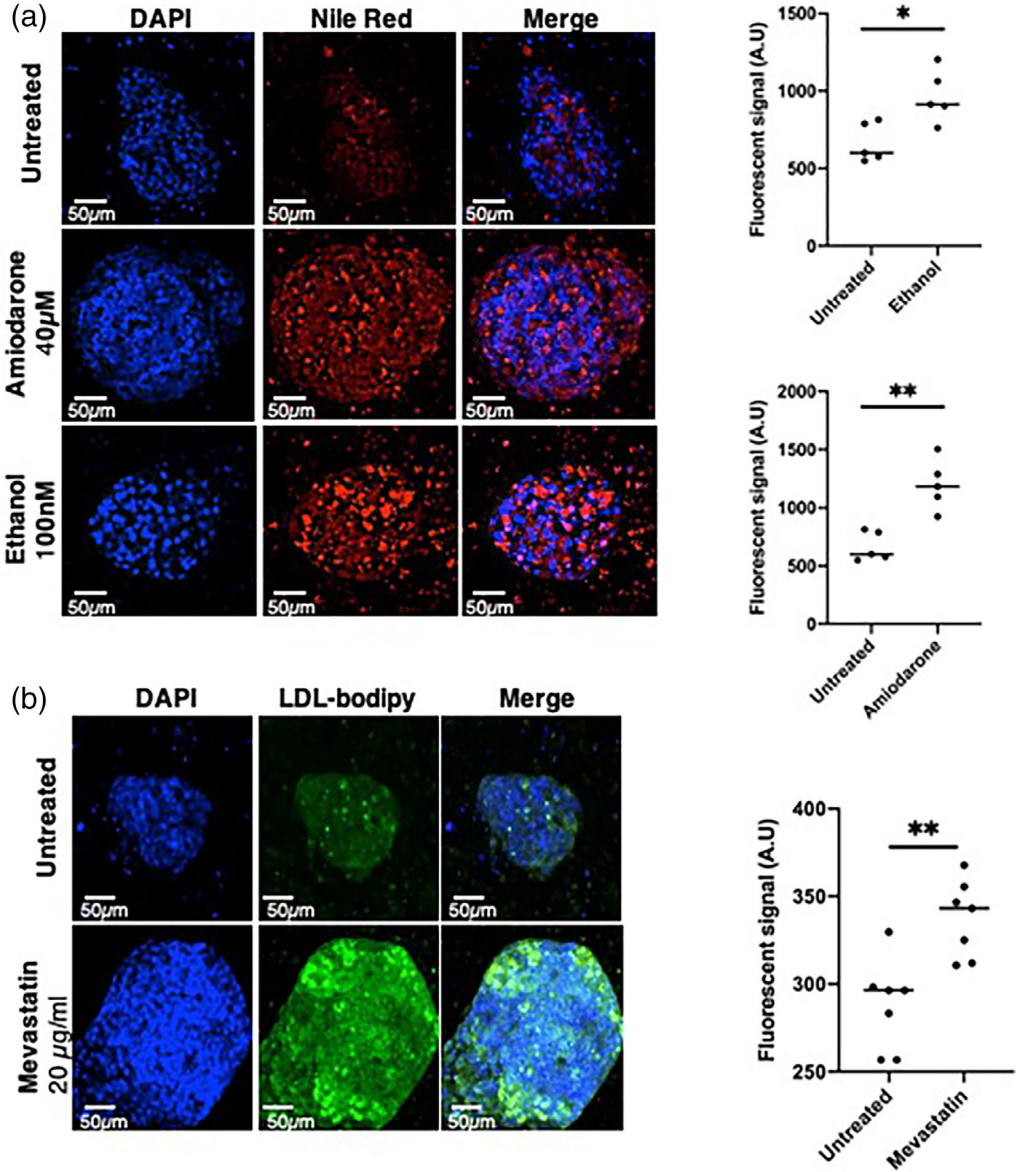
Lipid metabolism in liver organoids. (a) *Left panel*, liver organoids stained with DAPI (blue, nuclei) and Nile red (red, lipid droplets) in untreated conditions, and upon amiodarone (40 μM) or ethanol (200 nM) 24 h treatments. *Right panel*, fluorescence quantification (*N* = 5). (b) *Left panel*; liver organoids incubated with LDL‐bodipy (green) in untreated condition and upon mevastatin treatment. Nuclei were stained with DAPI (blue). *Right panel*, fluorescence quantification (*N* = 7). Statistical significance was assessed using unpaired *t* test, with a *p* value cut‐off set at *p* < 0.05. *, *p* value <0.05; **, *p* value <0.01; ***, *p* value <0.001; ns, not significant.

To assess the functionality of liver organoids regarding lipid metabolism, we tested their ability to store and internalize lipids using amiodarone‐induced steatosis^28^ and ethanol‐induced lipid biosynthesis^29^ treatments. Nile‐red staining revealed a significant higher accumulation of lipids in liver organoids after exposure to both amiodarone and ethanol when compared to untreated organoids (Figure 7a). Then, we assessed the ability of liver organoids to internalize low‐density lipoprotein (LDL) particles, as well as their ability to respond adequately to statin treatment. As shown in Figure 7b, liver organoids were able to uptake LDL particles and a preincubation with mevastatin significantly increased the LDL internalization.

#### 2.5.3 Apo(a) production

**FIGURE 8:**
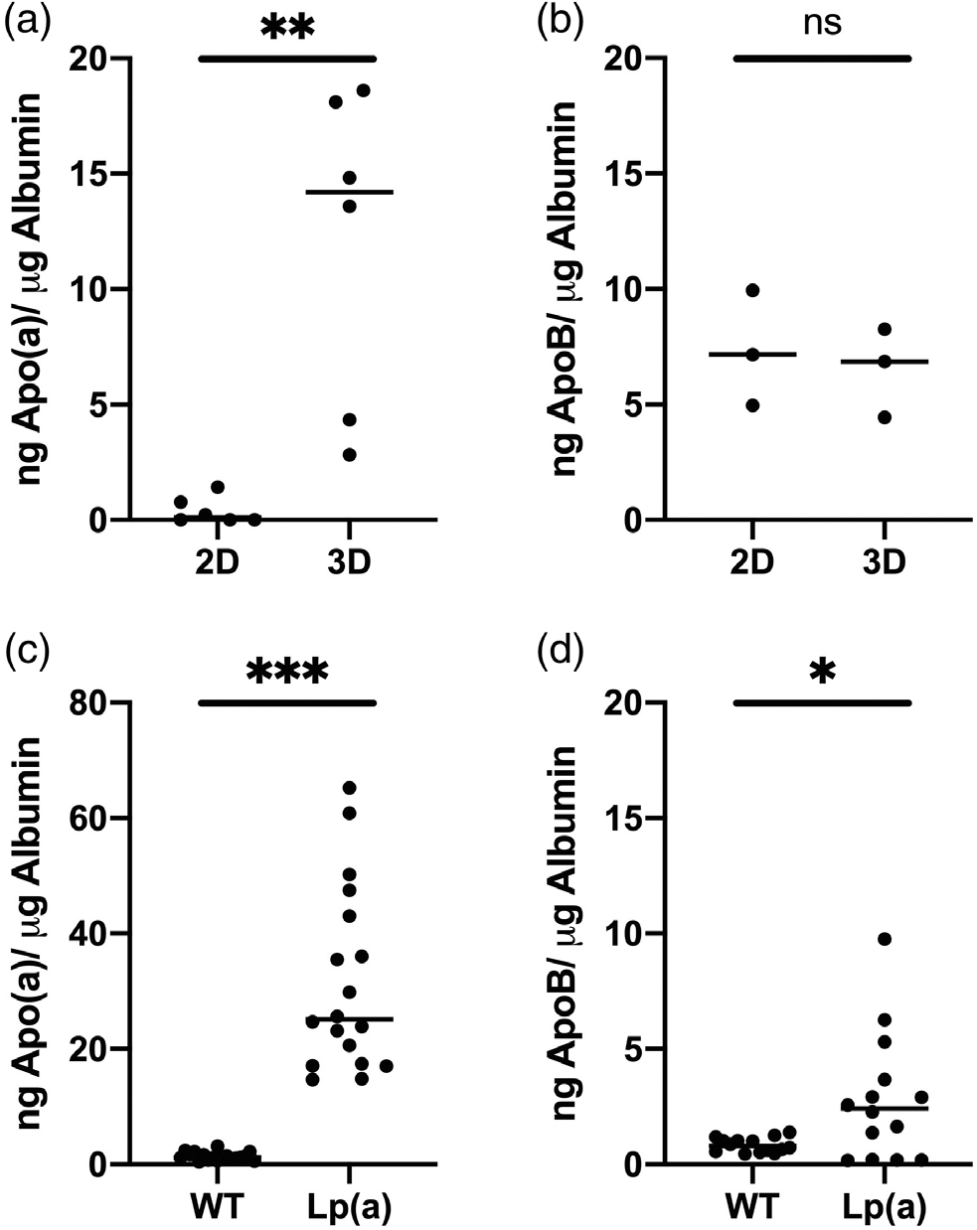
Extracellular levels of Apo(a) (a) and ApoB (b) in 24 h medium of hyper‐Lp(a) hepatocyte‐like cells (HLCs) (2D) (*n* = 6) and liver organoids (3D) (*n* = 3). Extracellular levels of Apo(a) (*n* = 18) (c) and ApoB (n = 14) (d) in 48 h medium of control (WT) and hyper‐Lp(a) (Lp(a)) liver organoids. Values are normalized against albumin production. Statistical significance was assessed using unpaired *t* test, with a *p* value cut‐off set at *p* < 0.05. *, *p* value <0.05; **, *p* value <0.01; ***, *p* value <0.001; ns, not significant.

The production of native Apo(a) was tested in the supernatant of liver organoids and HLCs derived from hiPSC from two distinct patients: (i) a control patient and (ii) a patient carrying genetically high levels of plasma Lp(a).^17^ While undetectable in HLCs (2D), the production of Apo(a) for the control patient was detected in liver organoids (3D) with a mean concentration at 0.24 ng/μg albumin in 24 h (Supplemental Figure 5). For the patient with high Lp(a) concentrations, traces of Apo(a) were measured in 2D at a mean concentration of 0.42 ng/μg albumin but increased up to 50× folds using the 3D model with a mean concentration at 21.1 ng/μg albumin in 24 h (Figure 8a). In comparison, the secretion of ApoB was similar between the 2D and 3D models (Figure 8b). When the secretion time was increased to 48 h, the accumulation of extracellular levels of Apo(a) remained higher in the patient with high levels of Lp(a) compared to the control patient, with, respectively, a mean of 31.5 and 1.3 ng/μg albumin (Figure 8c). The concentration of ApoB in the supernatant was also significantly increased in the supernatant of liver organoids derived from the patient with high Lp(a) concentrations compared to control (Figure 8d).

## 3 DISCUSSION

We generated and characterized a novel functional 3D liver model derived from hiPSCs using Biomimesys® as a matricial microenvironment. From a translational perspective, this new 3D protocol has the advantage to offer better functionality than the classic 2D cultured HLC‐models. In particular, it offers the possibility to study Lp(a) metabolism which was restricted to PHH so far. Thus, this model represents an interesting tool for the functional characterization, as well as for drug screening of new therapeutic targets, in the field of dyslipidemia.

PHH are considered to be the reference tool for studying different liver functions in vitro, but also to develop new drugs and evaluate their toxicity. However, the availability of these cells is limited due to the small number of donors. Moreover, they present with limitation related to their stability and functionality ex vivo. The development of protocols aiming at differentiating hiPSC into liver cells has, however, provided an almost inexhaustible source of hepatocytes.^14^ In particular, we^3^ and others,^30^ have developed models of hiPSCs differentiated into HLCs to study the metabolism of lipoproteins. A known limitation of these models is that HLCs keep residual characteristics of fetal or neonatal hepatocytes, with persistent expression of alpha‐fetoprotein and low albumin production.^4^ This immaturity of HLCs could be explained by the fact that hepatocyte functions require a well‐defined spatial organization of the cells, which is impossible to reproduce in culture as a cellular monolayer. On the contrary, 3D culture approaches, particularly organoids, allow the generation of more complex structures that can reproduce the 3D organization of tissues. Recently, protocols for the differentiation of iPSCs into liver organoids have been developed to obtain different cell types that make up the liver and demonstrate the high potential of these models for the study of complex metabolic diseases relying on the interactions of several cell types.^10^, ^31^

Several techniques have been developed for the formation of organoids from stem cells or differentiated cells, relying on the self‐assembly capacity of the cells or on the use of preformed or printed ECM.^32^, ^33^, ^34^ The adhesion of cells and their homeostasis within the organoids are consolidated by the ECM that allows exchanges between the cells and plays a role in the physical support of the 3D structure. This ECM can be endogenous, that is, produced by the cells within the organoid,^35^ or exogenous.^36^ In the study described herein, the organoids were differentiated from the beginning within a hydroscaffold for providing anchor and matricial clues to the iPSCs. The grown organoids were shown to produce their own collagen in the surroundings, showing that both the exogenous and endogenous matrix can coexist.

The first hiPSC‐based platforms for modeling metabolic diseases, such as NAFLD/NASH, involved the differentiation and the coculture in hydrogel of up to four different cell types: HLCs, hepatic stellate cells, macrophages/Kupffer cells, and endothelial cells^37^; a process that limits the scaling up for high throughput screening strategies.^31^ One of the strengths of our human liver organoid model is the fact that it can generate several cell types that self‐aggregate within the matrix from a single hiPSC line. Indeed, transcriptomic analyses identified our model as a multi‐tissue organoid composed of parenchymal (hepatocytes and cholangiocytes) and non‐parenchymal (endothelial and stellate cells) cells found in the liver. This liver‐like cellular repertoire was further confirmed using immunostaining. Additional experiments such as single cell analysis are required in order to confirm this repertoire and give a semiquantitative estimation of the proportion for each cell type as described previously.^10^, ^38^ The availability of such a multi‐tissue model will open new perspectives for its application in the study of complex metabolic diseases such as NAFLD/NASH.^10^ Moreover, we described that this 28‐days protocol allows the present model to be used for up to 35 days, opening perspectives for chronic toxicological tests and long‐term challenge studies.

One of the goals of developing such hiPSC‐derived models in 3D environment is to enhance the functionality and notably to limit as much as possible the “fetal‐like” phenotype of HLC derived from hiPSC. We tested the model for its functionality with a special emphasis on lipid and drug metabolism. We first identified that the model produces active cytochrome oxydases (CYP3A4, 1A2, 2C9, 2D6, 2B6) and we confirmed that the hIPS‐derived organoids display an overall improved CYP activity compared to the 2D cultured HLCs, as previously described.^38^ Second, in order to model the first stage of NAFLD, we grew the organoids under pro‐steatogenic culture conditions and confirmed their ability to store lipids. Additional analyses are underway to understand the molecular mechanisms underlying the development of steatosis in our model, in particular lipid uptake capacity, as well as lipid biosynthesis (de novo lipogenesis) and secretion pathways. Additional studies are also required to test the suitability of the model for modeling a lipid‐induced inflammation and fibrosis in a perspective of MASH studies.^10^ Moreover, availability of standardized procedures for genome engineering in hiPSCs^39^ opens new perspective for the use of the present model for CRISPR‐based target discovery and drug screening for steatosis. Indeed, Hendriks et al.^40^ recently used engineered human fetal hepatocyte organoids for hepatic steatosis modeling and identified that compounds repressing de novo lipogenesis were the most efficient in reducing steatosis.

While the production of apolipoproteins (APOA1, APOA4, APOC3, APOD) was previously reported in an hiPS‐derived organoid model,^38^ our model is the first able to produce apo(a). This finding is of great translational relevance since such ability to secrete apo(a) is being restricted to PHH up to now. Availability of an apo(a) producing model will help understanding the metabolism of Lp(a) which remains unclear so far, notably regarding the regulation of hepatic apo(a) production by PCSK9.^41^ It will also allow to perform drug screening to reduce Lp(a) secretion, which is one of the last remaining frontiers in the management of dyslipidemia.^42^ Recent studies suggested that sex may play a role in the functionality and response to pharmacological challenges in iPSCs.^43^ It is important to note that our model was derived from a male donor and that our results should be confirmed on iPSCs derived form a female donor (also with an elevated Lp(a) concentration).

Improvements of the model to further enhance its functionality are potentially feasible. For instance, it is possible to embed the hydroscaffold and the hiPSC‐derived organoids into a microfluidic device, as previously described with HepG2 cells grown in Biomimesys® with enhanced functionalities.^44^

## 4 MATERIALS AND METHODS

### 4.1 hiPS cell culture

Each subject entering the study agreed to and signed an institutional review board‐approved statement of informed consent for the collection of urine samples and their use thereof (authorization number from the French Ministry of Health: DC‐2011‐1399).

The ITXi001‐A hiPSC line was reprogrammed from a 46‐year‐old woman and characterized previously.^3^ It was used for all the steps of the protocol setup and liver organoids characterization. For apo(a) production, we used the ITXi0012‐A hiPSC line reprogrammed from a 28 years‐old man carrying a hyperlipoprotein(a)emia^17^ and recently characterized.^45^ HiPSCs were cultured on plates coated with 0.05 mg/ml Matrigel (Corning) in StemMACS™ iPS‐Brew XF medium (Miltenyi) and passages were performed using the Gentle Cell Dissociation Buffer (Stem Cell Technologies).

### 4.2 Differentiation of hiPSCs into liver organoids

To induce 3D differentiation, hiPSCs were seeded in 96‐well Biomimesys® *Liver* plates at 100,000 cells/well in 10 μl/well of StemMacs™ iPS Brew XF medium supplemented with 10 μM ROCK inhibitor (Y27632, Cell Guidance Systems). After seeding, hiPSCs were incubated for 10 min at 37°C under hypoxia (4% O_2_, 5% CO_2_) and 190 μl/well (QSP 200 μl/well) of StemMacs™ iPS Brew XF medium supplemented with 10 μm ROCK inhibitor (Cell Guidance Systems) was added in each well. The cells were then incubated under hypoxia (4% O_2_, 5% CO_2_) for 3 days before starting the differentiation protocol at day 0. The sequential steps to achieve the differentiation of hiPSCs toward liver organoids are listed in Table 1.

All medium changes were performed using a multichannel pipet by removing 100 μl/well medium before adding 100 μl/well fresh medium. Functional experiments were performed up to 3 days after the end of the differentiation protocol, unless otherwise specified.

### 4.3 Synthesis of Biomimesys® *Liver* hydroscaffold

The HA‐based hydroscaffold™ Biomimesys® *Liver* was developed by HCS Pharma (Loos, France) and was synthesized in three steps. The first step consisted in the modification of HA (ACROS, Belgium) using RGDS (HA‐g‐RGDS) as described previously.^46^ The second step consisted in the modification of HA using Galactosamine (GalN). The HA (2 g, 5 mmol) was dissolved in water to a concentration of 2 g/L and GalN hydrochloride (TCI Europe, Belgium) (100 mg, 0.46 mmol) was added to the solution where the pH was adjusted and maintained at 4.75 by continuous adding of 1 M HCl (Fisher Scientific, Illkirch, France). *N*‐(3‐dimethylaminopropyl)‐*N*‐ethylcarbodiimide hydrochloride (EDCI, TCI Europe, Belgium) (1.2 g, 6.25 mmol) and *N*‐hydroxysuccinimide (NHS, TCI Europe, Belgium) (0.1 g, 0.8 mmol) were then added and the reaction was allowed to proceed for 12 h at room temperature. The polymer solution was dialyzed against acidic, then alkaline, and finally pure water, before being concentrated, and freeze‐dried to give the HA derivative, denoted HA grafting Galactosamine (HA‐g‐GalN).

The last step consisted in crosslinking HA‐g‐RGDS, HA‐g‐GalN, type I, and Type IV collagens. Briefly, a mix of HA‐g‐RGDS (50%) and HA‐g‐GalN (50%) was completely dissolved in pure water before adding 7.22 μmol of type I collagen (Santa Cruz, USA) and 2 μmol type IV collagen (Sigma Aldrich, USA), and let stirred for 1 h to obtain a homogenous solution. Final hydrazide cross‐linker (ADH, TCI Europe, Belgium) was dissolved in milliQ‐water and added to the solution, before adjusting the pH to 4.75 with 1 M HCl. The carbodiimide reagent (EDCI) was dissolved in milliQ‐water, added to the reaction mixture and allowed to gel for 2 h with gentle agitation. Hydrogels were dialyzed against 0.1 N NaCl, then in a water:ethanol mixture (3:1, v/v), and in milliQ‐water to remove unreacted ADH and EDCI. The purified hydrogel was cast in 96‐well plates and frozen, before being placed in a freeze dryer during 24 h (Crios, Cryotec, France; performances, 3 kg ice/24 h, T = −55°C) and finally sterilized with UV irradiation.

### 4.4 SEM observations

For the observation of empty hydroscaffold™ (no cells added), three washes were performed in ultrapure water, then samples were frozen in liquid nitrogen (cryo‐fixation) and immediately lyophilized (Crios, Cryotec, France; performances, 3 kg ice/24 h, *T* = −55°C). For cell‐containing hydroscaffolds™, samples were fixed in 2.5% glutaraldehyde at 4°C during 2 h, rinsed, frozen, and lyophilized. All samples were sputter‐coated with gold (Polaron) and examined on a SEM (EVO 40 EP ZEISS Zeiss, Germany). Pore size measurement in the empty hydroscaffolds was determined using Image J software.

### 4.5 RNA extraction

RNA samples were extracted using the NucleoSpin Tissue Purification Kit (MACHEREY‐NAGEL). Prior to extraction, liver organoid‐containing hydroscaffolds™ were transferred into an Eppendorf tube with RA1 lysis buffer (NucleoSpin Tissue Purification Kit), then dissected using a 23G needle in order to break the hydroscaffold and increase the yield of RNA recovery.

### 4.6 Transcriptomic analysis

3′‐Digital gene expression profiling protocol was performed as previously described.^47^, ^48^ Briefly, the libraries were prepared from 10 ng of total RNA. RNA samples were extracted from three different differentiations of hiPSC into liver organoids at different time points (days 0, 2, 5, 7, 10, 13, 16, 19, 22, 25, 28, and 49). The poly(A) tail was tagged with universal adapters, well‐specific bar‐codes and unique molecular identifiers (UMIs) during template‐switching reverse transcriptase. Barcoded cDNAs from multiple samples were then pooled, amplified, and fragmented using a transposon‐fragmentation approach, which enriches for 3′ends of cDNA. Then, 100 ng of full‐length cDNAs was used as input to the Nextera DNA Sample Prep kit (ref FC‐121‐1030, Illumina), which enriches for 3′ends of cDNA. The length of library DNA fragments was controlled on 2200 Tape Station System (Agilent Technologies). A library of 350–800 bp was run on an Illumina HiSeq 2500 using a HiSeq Rapid SBS Kit v2 (50 cycles; FC‐402‐4022) and a HiSeq Rapid PE Cluster Kit v2 (PE‐402‐4002) according to manufacturer's protocol (Denaturing and Diluting Libraries for the HiSeq® and GAIIx, Part # 15050107 v03 protocol, Illumina). Raw fastq pairs used for analysis matched the following criteria: all 16 bases of the first read had quality scores of at least 10, and the first six bases correspond exactly to a designed well‐specific barcode.

The second read (58 bases) corresponds to the captured poly(A) RNAs sequence. We performed demultiplexing of these fastq pairs in order to generate one single‐end fastq for each of the samples. These fastq files are then aligned with bwa to the reference mRNA refseq sequences and the mitochondrial genomic sequence, both available from the UCSC download site. Gene expression profiles were generated by parsing alignment files (.bam) and counting for each sample the number of UMIs associated with each gene. Reads aligned on multiple genes, containing more than three mismatches with the reference sequence or having a polyA pattern were discarded. Finally, a matrix containing the counts of all genes on all samples is produced. The expression values, corresponding to the absolute abundance of mRNAs in all samples, were then ready for further gene expression analysis. The R package deseq2^49^ is then used for the differential analysis.

### 4.7 Functional assessment of lipid metabolism

In order to test the ability of liver organoids for lipid biosynthesis and accumulation, 28 days liver organoids were exposed for 2 days with 40 mM amiodarone, 100 nM ethanol and 0.1% DMSO for control. After fixation of the cells with paraformaldehyde (PFA 4% in PBS) for 15 min at room temperature, the lipids were labeled with Nile Red (50 ng/ml) for 1 h at room temperature, washed three times with PBS (1000 μl) and processed for imaging.

In order to test the capacity of the model to uptake LDL and its response to drug treatment, liver organoids were first exposed for 20 h with DMSO (control) or with 20 mg/ml mevastatin (Sigma) followed by 3 h incubation with LDL‐bodipy (Invitrogen™). Once the incubation with LDL‐bodipy was completed, the scaffolds were washed three times with HCM medium (LONZA) at 37°C and fixed with PFA (4% in PBS) for 15 min at room temperature before imaging.

### 4.8 ELISA for Apo(a), ApoB, and albumin

Extracellular levels of Apo(a) in culture supernatants were directly measured using the human Apo(a) ELISA Kit (Cell Biolabs, STA‐359) following the manufacturer's instructions.

For ApoB ELISAs (MabTech, 3715‐1H‐6), experiments were performed using 96‐wells MaxiSorp plates (ThermoScientific) following the manufacturer protocol and using culture supernatants diluted 3 and 10× in PBS containing 0.05% Tween 20 and 0.1% BSA (Sigma, A7030) for liver organoids and HLCs, respectively. For albumin ELISAs (R&D systems, DY1455), experiments were performed according to the manufacturer's protocol and using culture supernatants diluted X5 in PBS containing 1% BSA (Sigma, A7030). Absorbance was read using a Varioskan Microplate reader (ThermoScientific).

### 4.9 Immunofluorescence

Samples were fixed in PFA 4% for 15 min, embedded in paraffin wax with Excelsior ES (ThermoScientific) and processed into 4 μm thickness section.

For immunofluorescence staining, paraffin and antigen retrieval was performed on rehydrated sections using citrate buffer (citric acid at 0.1 M and sodium citrate at 0.1 M) for 20 min at 95°C. Sections were then pretreated for unspecific binding using a 30 min incubation at RT with a PBS solution containing 10% normal goat serum. Hydrophobic pen was applied around to section before an overnight incubation with primaries antibodies at 4°C (Table 2). Slides were further washed using PBS and incubated for 1 h at RT with appropriate Alexa‐Fluor‐conjugated secondary antibodies (Table 2).

**TABLE 2:** List and dilution of primary and secondary antibodies.

Antibody	Provider	Reference	Dilution
Albumin	Tebu‐bio	CL2513A	1/200
Desmin	Abcam	ab32362	1/500
CFTR	Invitrogen	MA1‐935	1/400
CD31	Abcam	ab24590	1/200
ZO1	Invitrogen	33‐9100	1/100
MRP2	Abcam	ab172630	1/200
Anti‐Mouse Alexa Fluor 647	Ozyme	4410S	1/1000
Anti‐Rabbit Alexa Fluor 488	Ozyme	4412S	1/1000

Finally, sections were mounted with Mowiol® mounting medium with Hoechst for nuclei staining (SIGMA).

For imaging, we used whether the ImageXpress Micro Confocal system (Molecular Devices) for fluorescence or a bi‐photonic microscope (Nikon) for fluorescence and collagen I detection in liver organoids. Image analysis was performed using ImageJ (Nikon).

### 4.10 Statistical analysis

Data are expressed as mean ± SD. Significant differences between mean values were determined with the Mann–Whitney *U* test for comparison of two groups or paired Student's *t* test if appropriate. For the cluster approach, genes belonging to the same biological function or cell type are known to exhibit correlated expression. We used hierarchical clustering to detect groups of correlated genes supported by a statistical method (limma) to detect differential expression among biological conditions.

## 5 CONCLUSION

To conclude, this new hiPSC‐derived liver organoid model is a relevant tool to study in depth human lipoprotein metabolism and to sustain drug discovery strategy in the field of dyslipidemia and metabolic diseases.

## AUTHOR CONTRIBUTIONS

**Meryl Roudaut:** Conceptualization; formal analysis; investigation; methodology; validation; visualization; writing – original draft; writing – review and editing. **Amandine Caillaud:** Conceptualization; formal analysis; investigation; methodology; validation; visualization; writing – original draft; writing – review and editing. **Zied Souguir:** Conceptualization; investigation; methodology; visualization. **Lise Bray:** Investigation. **Aurore Girardeau:** Investigation. **Antoine Rimbert:** Formal analysis; validation; visualization; writing – review and editing. **Mikaël Croyal:** Formal analysis; investigation; methodology. **Gilles Lambert:** Funding acquisition; resources; writing – review and editing. **Murielle Patitucci:** Investigation. **Gaspard Delpouve:** Investigation. **Élodie Vandenhaute:** Writing – original draft; writing – review and editing. **Cédric Le May:** Funding acquisition; supervision; writing – review and editing. **Nathalie Maubon:** Conceptualization; funding acquisition; project administration; supervision; writing – original draft. **Bertrand Cariou:** Conceptualization; funding acquisition; project administration; supervision; writing – original draft; writing – review and editing. **Karim Si‐Tayeb:** Conceptualization; formal analysis; funding acquisition; investigation; methodology; project administration; supervision; validation; visualization; writing – original draft.

## CONFLICT OF INTEREST STATEMENT

B.C. has received research funding from Amgen, Pfizer, Sanofi, and Regeneron Pharmaceuticals Inc outside of the present work; and has served on scientific advisory boards and received honoraria or consulting fees from Amgen, Astra‐Zeneca, Eli‐Lilly, Novartis, Pfizer, and Sanofi. Z.S., E.V., G.D. M.R., and N.M. are employees of HCS Pharma. N.M. owns stocks in HCS Pharma. The other authors declare no conflict of interest.

## Supporting information


**DATA S1:** Supporting Information.

**SUPPLEMENTAL FIGURE 1:** Expression of pluripotency (*OCT4*, *NANOG*), hepatocyte specific (*HNF4*, *ALB*, *APOB*), or liver cell‐type specific (*SOX9*, *LYVE1*, *RBP1*) genes assessed by RT‐qPCR.

**SUPPLEMENTAL FIGURE 2:** RNA sequencing comparison of hiPS undifferentiated and differentiated into hepatocyte‐like cells (2D) versus liver organoids (3D). A. *Left Panel*, diagram depicting the comparison performed; *right panel*, Heatmap displaying differentially expressed genes between undifferentiated cells in 2D and 3D and differentiated cells into hepatocyte‐like cells and liver organoids (n = 3) independent differentiation, p < 0.05; *bottom panel*, diagram depicting the sample‐to‐sample distance. B. Volcano plot showing 7448 genes differentially expressed among 57,905 total genes between hepatocyte‐like cells and liver organoids.

**SUPPLEMENTAL FIGURE 3:** Gene ontology analysis of differentially expressed genes between 2D and 3D models.

**SUPPLEMENTAL FIGURE 4:** 3′SRP transcriptional analysis of apoptotic genes during the differentiation of liver organoids.

**SUPPLEMENTAL FIGURE 5:** Extracellular levels of Apo(a) in 24 h medium of control HLCs (2D) and liver organoids (3D). Values are normalized against albumin production. Statistical significance was assessed using unpaired t‐test, with a p value cut‐off set at p < 0.05. *, p value <0.05; **, p value <0.01; ***, p value <0.001; ns, not significant.

## Data Availability

The data that support the findings of this study are available from the corresponding author upon reasonable request.
