# Supplementary material for: Human induced pluripotent stem cells‐derived liver organoids grown on a Biomimesys® hyaluronic acid‐based hydroscaffold as a new model for studying human lipoprotein metabolism
Source: Bioeng Transl Med. 2024 Mar 16;9(4):e10659. doi: 10.1002/btm2.10659 (PMC11256179; doi:10.1002/btm2.10659)
Supplement: Supplementary file 1 — DATA S1: Supporting Information. [file BTM2-9-e10659-s003.docx]

**Supplemental Materials and Methods**

hiPSC differentiation into HLCs

HiPSCs were differentiated into HLCs as previously described^15^. Briefly, once cells reach ~70-80% confluency, hiPSCs were cultured in RPMI 1640 medium (Life Technologies) supplemented with B27 (with insulin) (Life Technologies), Activin A 100 ng/ml (Miltenyi), FGF2 20 ng/ml (Miltenyi) and BMP4 10 ng/ml (Miltenyi) for 2 days in normoxia (20% O2, 5% CO2), then switched to RPMI 1640 with Activin A 100 ng/ml for three days to induce definitive endoderm (DE) cells. DE cells were further differentiated into hepatic progenitor cells for 5 days in RPMI supplemented with BMP4 20 ng/ml and FGF2 10 ng/ml in hypoxia (4% O_2_, 5% CO_2_). Then, cells were cultured for 5 days as immature hepatocytes in RPMI 1640 supplemented with HGF 20 ng/ml (Miltenyi) under hypoxia (4% O_2_, 5% CO_2_). Then, cells were directed into mature hepatocytes using hepatocyte culture medium (HCM) (Lonza) supplemented with OSM 20 ng/ml (Miltenyi) for additional 5-6 days under normoxia (20% O_2_, 5% CO_2_). RNA samples were collected every other day of the differentiation (day 0 to day 20) and processed further for gene expression analysis.

**Rheological measurements**

Rheological properties of Biomimesys^®^ *Liver* were measured on a Discovery HR-2 rheometer (Thermal Analysis Instruments, France) equipped with 8 mm parallel plate geometry on a stage heated to 37°C. Hydroscaffold were tested at a frequency of 1 Hz and a logarithmic sweep from 1 to 500 Pa with 5 points per decade. The shear storage modulus was determined by averaging at least 5 points in the linear viscoelastic region (LVR). The shear modulus measurements taken with the rheological measurements was converted to an elastic modulus E (KPa) value using the following equation E = 3 G. The swollen hydrogels were then gently drained to remove the excess medium before being weighed (mw). The swelling ratio (g/g) was measured by dividing the weight of the swollen hydrogel by the weight of the initial dried sample (md) according the equation Q = (mw –md)/md.

**Assessment of CYP450 activity by mass spectrometry**

Liver organoids were treated with different substrates (SIGMA) specific to a given CYP (CYP1A2: phenacetin (50 µM); CYP3A4: testosterone (50 µM); CYP2B6: bupropion (100 µM); CYP2C9: diclofenac (20 µM); and CYP2D6: dextromethorphan (20 µM)) in the presence of salycilamide (3 mM), a phase 2 enzyme inhibitor. A control condition (namely “basal”) is performed with DMSO 0,1% instead of inducer. After 4 hours incubation, the cell supernatant was recovered in acetonitrile (1/1 v:v) and the cell pellet was kept for further RNA extraction for normalization  (-80°C).

Concentrations of CYP-dependent products (CYP1A2: phenacetin → acetaminophen; CYP3A4: testosterone → 6b-hydroxytestosterone; CYP2B6: bupropion → 6-hydroxybupropion; CYP2C9: diclofenac → 4’-hydroxydiclofenac; and CYP2D6: dextromethorphan → dextrorphan) were determined using liquid chromatography-tandem mass spectrometry (LC-MS/MS) analysis. All solvents used were LC-MS grade and purchased from Biosolve (Valkenswaard, Netherlands). Standard compounds were obtained from Sigma Aldrich (Saint-Quentin Fallavier, France). A pool of reference standard solutions was prepared and serially diluted in acetonitrile to obtain seven standard solutions ranging from 0.01 to 1.0 µmol/L. A pool solution of exogenous internal standards (*D_4_*-acetaminophen, *D_3_-*testosterone, *D_6_-*6-hydroxybupropion, *^13^C_6_*-4’-hydroxydiclofenac and *D_3_-*dextrorphan) was prepared at 0.2 µmol/L in acetonitrile and was added (400 µL) to cell supernatants (400 µL) and standard solutions (400 µL). Samples were then vortex-mixed and dried under a gentle stream of nitrogen. Dried samples were finally reconstituted with 100 µL of 25% acetonitrile. Analyses were performed on a Xevo® TQD mass spectrometer with an electrospray interface and an Acquity H-Class® UPLC^TM^ device (Waters Corporation, Milford, MA, USA). Samples (5 µL) were injected onto a BEH-C_18_ column (1.7 µm, 2.1 × 50 mm, Waters Corporation) held at 60 °C. Compounds were separated using a linear gradient of mobile phase B (100% acetonitrile, 0.1% formic acid) in mobile phase A (5% acetonitrile, 0.1% formic acid) at a flow rate of 600 µL/min. Mobile phase B was kept constant for 0.5 min at 1%, linearly increased from 1% to 100% for 3 min, kept constant for 0.5 min, returned to the initial condition over 0.5 min, and kept constant for 0.5 min before the next injection. Targeted compounds were then detected by the mass spectrometer with the electrospray interface operating in the positive ion mode (capillary voltage, 3 kV; desolvatation gas (N_2_) flow and temperature, 900 L/h and 350 °C; source temperature, 150 °C). The multiple reaction monitoring mode was applied for MS/MS detection as detailed in supplemental Table S1. Chromatographic peak area ratios between unlabeled compounds and their respective internal standards constituted the detector responses. Standard solutions were used to plot calibration curves for quantification. The linearity was expressed by the mean r² which was greater than 0.998 for all compounds (linear regression, 1/x weighting, origin excluded). Data acquisition and processing were achieved using MassLynx® and TargetLynx® version 4.1 software (Waters Corporation).

**Supplemental Table S1.** Multiple reaction mode (MRM) transitions used for LC-MS/MS analysis of CYP450 activities.

| **CYP450** | **Compounds** | **MRM transition (*m/z*)** | **Cone / collision (V)** |
| --- | --- | --- | --- |
| CYP1A2 | Acetaminophen | 152.0 → 110.0 | 30 / 15 |
|  | *D_4_*-acetaminophen | 156.0 → 114.0 | 30 / 15 |
|  | Phenacetin | 180.0 → 110.0 | 30 / 35 |
| CYP2B6 | 6-hydroxybupropion | 256.1 → 238.0 | 30 / 15 |
|  | *D_6_*-6-hydroxybupropion | 262.1 → 244.1 | 30 / 15 |
|  | bupropion | 240.0 → 184.0 | 30 / 12 |
| CYP2D6 | Dextrorphan | 258.1 → 157.0 | 55 / 38 |
|  | *D_3_*-dextrorphan | 261.3 → 157.0 | 55 / 38 |
|  | Dextromethorphan | 272.2 → 147.0 | 50 / 30 |
| CYP3A4 | 6b-hydroxytestosterone | 305.1 → 269.2 | 45 / 15 |
|  | *D_3_*-testosterone | 308.2 → 272.2 | 45 / 15 |
|  | testosterone | 289.2 → 108.9 | 45 / 25 |
| CYP2C9 | 4’-hydroxydiclofenac | 312.0 → 230.0 | 30 / 25 |
|  | ^13^C_6_-4’-hydroxydiclofenac | 318.0 → 236.1 | 30 / 25 |
|  | Diclofenac | 296.0 → 214.0 | 30 / 25 |
