# Supplementary figures and images for: Human induced pluripotent stem cells‐derived liver organoids grown on a Biomimesys® hyaluronic acid‐based hydroscaffold as a new model for studying human lipoprotein metabolism
Source: Bioeng Transl Med. 2024 Mar 16;9(4):e10659. doi: 10.1002/btm2.10659 (PMC11256179; doi:10.1002/btm2.10659)

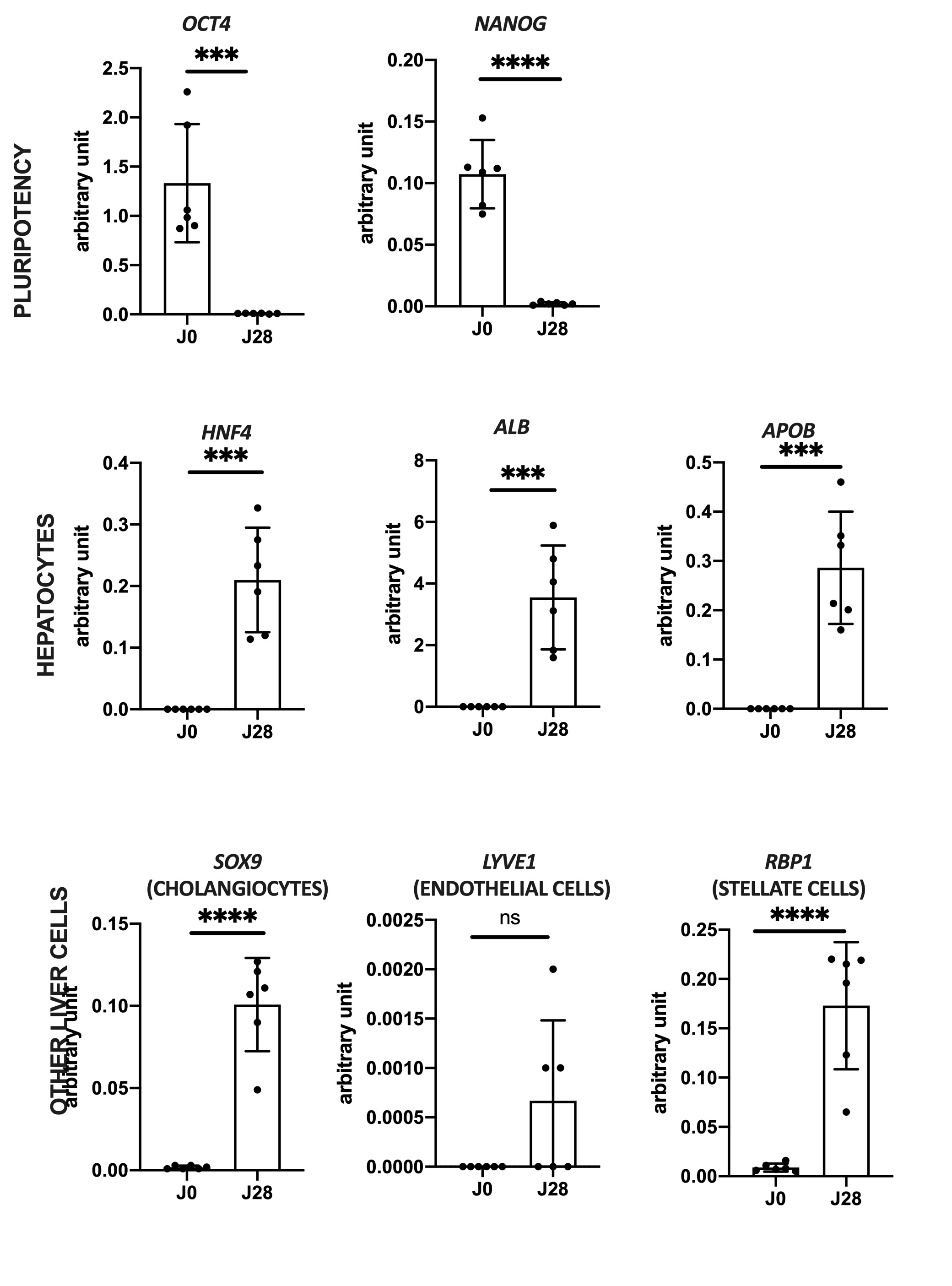

Supplement: Supplementary file 2 — SUPPLEMENTAL FIGURE 1: Expression of pluripotency (OCT4, NANOG), hepatocyte specific (HNF4, ALB, APOB), or liver cell‐type specific (SOX9, LYVE1, RBP1) genes assessed by RT‐qPCR. [file BTM2-9-e10659-s002.jpg]

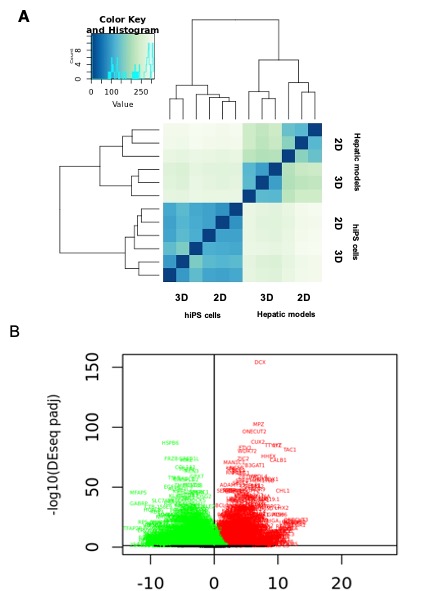

Supplement: Supplementary file 3 — SUPPLEMENTAL FIGURE 2: RNA sequencing comparison of hiPS undifferentiated and differentiated into hepatocyte‐like cells (2D) versus liver organoids (3D). A. Left Panel, diagram depicting the comparison performed; right panel, Heatmap displaying differentially expressed genes between undifferentiated cells in 2D and 3D and differentiated cells into hepatocyte‐like cells and liver organoids (n = 3) independent differentiation, p < 0.05; bottom panel, diagram depicting the sample‐to‐sample distance. B. Volcano plot showing 7448 genes differentially expressed among 57,905 total genes between hepatocyte‐like cells and liver organoids. [file BTM2-9-e10659-s006.jpg]

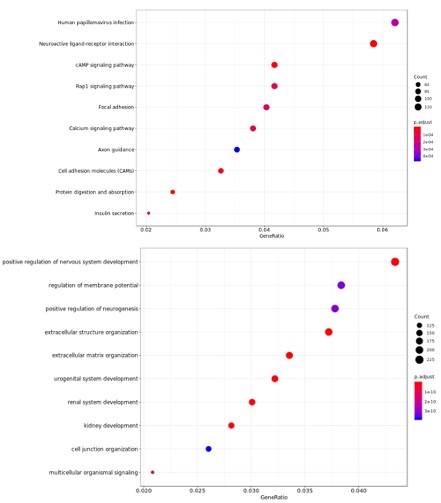

Supplement: Supplementary file 4 — SUPPLEMENTAL FIGURE 3: Gene ontology analysis of differentially expressed genes between 2D and 3D models. [file BTM2-9-e10659-s005.jpg]

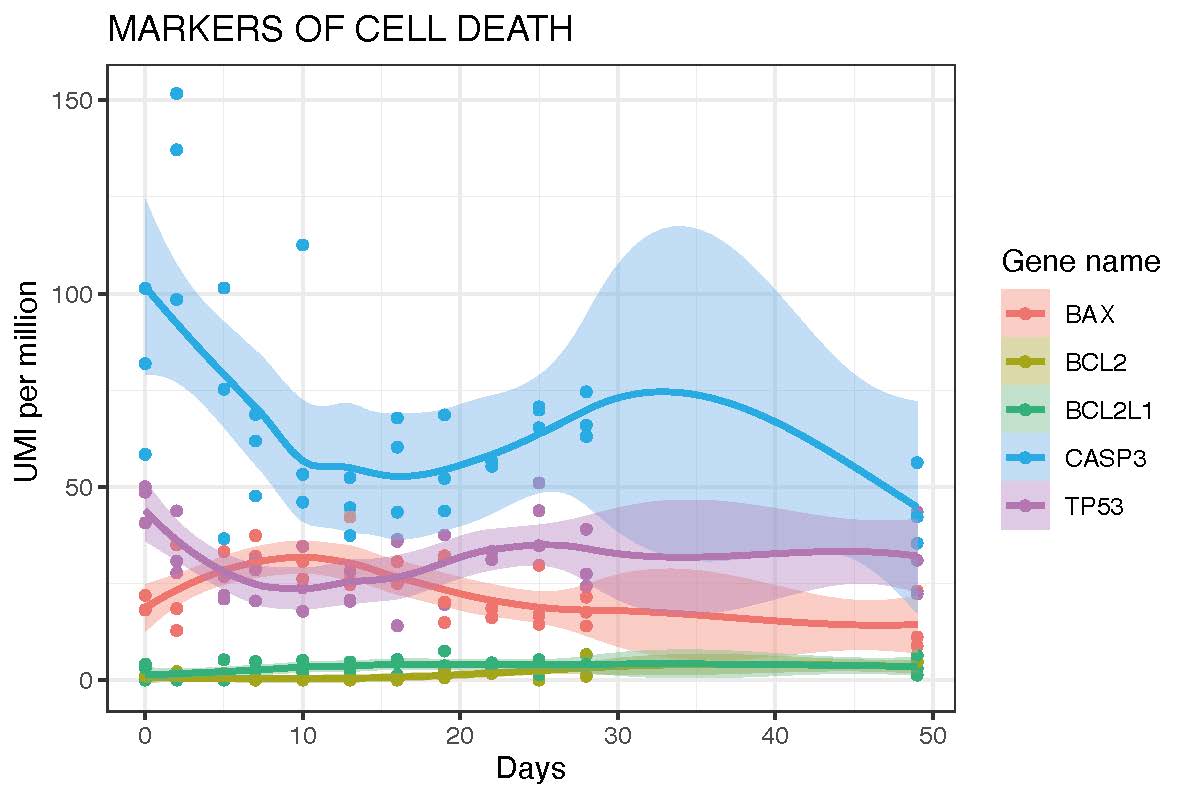

Supplement: Supplementary file 5 — SUPPLEMENTAL FIGURE 4: 3′SRP transcriptional analysis of apoptotic genes during the differentiation of liver organoids. [file BTM2-9-e10659-s001.jpg]

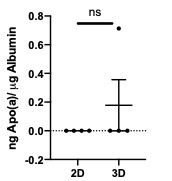

Supplement: Supplementary file 6 — SUPPLEMENTAL FIGURE 5: Extracellular levels of Apo(a) in 24 h medium of control HLCs (2D) and liver organoids (3D). Values are normalized against albumin production. Statistical significance was assessed using unpaired t‐test, with a p value cut‐off set at p < 0.05. *, p value <0.05; **, p value <0.01; ***, p value <0.001; ns, not significant. [file BTM2-9-e10659-s004.jpg]
